# The Intervention Nurses Start Infants Growing on Healthy Trajectories (INSIGHT) study

**DOI:** 10.1186/1471-2431-14-184

**Published:** 2014-07-18

**Authors:** Ian M Paul, Jennifer S Williams, Stephanie Anzman-Frasca, Jessica S Beiler, Kateryna D Makova, Michele E Marini, Lindsey B Hess, Susan E Rzucidlo, Nicole Verdiglione, Jodi A Mindell, Leann L Birch

**Affiliations:** 1 Department of Pediatrics, HS83, Penn State College of Medicine, 500 University Dr., Hershey 17033, PA, USA; 2 Public Health Sciences, Penn State College of Medicine, Hershey, PA, USA; 3 Center for Childhood Obesity Research, Penn State College of Health and Human Development, University Park, PA, USA; 4 Friedman School of Nutrition Science and Policy, Tufts University, Boston, MA, USA; 5 Biology, Eberly College of Sciences, Penn State University, University Park, PA, USA; 6 Surgery, Penn State College of Medicine, Hershey, PA, USA; 7 Psychology, Saint Joseph’s University, Philadelphia, PA, USA; 8 Department of Foods and Nutrition, University of Georgia, Athens, GA, USA

**Keywords:** Obesity, Prevention, Infancy, Responsiveness, Home visitation, Feeding, Parenting

## Abstract

**Background:**

Because early life growth has long-lasting metabolic and behavioral consequences, intervention during this period of developmental plasticity may alter long-term obesity risk. While modifiable factors during infancy have been identified, until recently, preventive interventions had not been tested. The Intervention Nurses Starting Infants Growing on Healthy Trajectories (INSIGHT). Study is a longitudinal, randomized, controlled trial evaluating a responsive parenting intervention designed for the primary prevention of obesity. This “parenting” intervention is being compared with a home safety control among first-born infants and their parents. INSIGHT’s central hypothesis is that responsive parenting and specifically responsive feeding promotes self-regulation and shared parent–child responsibility for feeding, reducing subsequent risk for overeating and overweight.

**Methods/Design:**

316 first-time mothers and their full-term newborns were enrolled from one maternity ward. Two weeks following delivery, dyads were randomly assigned to the “parenting” or “safety” groups. Subsequently, research nurses conduct study visits for both groups consisting of home visits at infant age 3–4, 16, 28, and 40 weeks, followed by annual clinic-based visits at 1, 2, and 3 years. Both groups receive intervention components framed around four behavior states: Sleeping, Fussy, Alert and Calm, and Drowsy. The main study outcome is BMI z-score at age 3 years; additional outcomes include those related to patterns of infant weight gain, infant sleep hygiene and duration, maternal responsiveness and soothing strategies for infant/toddler distress and fussiness, maternal feeding style and infant dietary content and physical activity. Maternal outcomes related to weight status, diet, mental health, and parenting sense of competence are being collected. Infant temperament will be explored as a moderator of parenting effects, and blood is collected to obtain genetic predictors of weight status. Finally, second-born siblings of INSIGHT participants will be enrolled in an observation-only study to explore parenting differences between siblings, their effect on weight outcomes, and carryover effects of INSIGHT interventions to subsequent siblings.

**Discussion:**

With increasing evidence suggesting the importance of early life experiences on long-term health trajectories, the INSIGHT trial has the ability to inform future obesity prevention efforts in clinical settings.

**Trial registration:**

NCT01167270. Registered 21 July 2010.

## Background

Overweight and rapid weight gain during infancy are associated with increased later risk of overweight [1-24], as well as numerous co-morbidities including hypertension [25-28], coronary heart disease [29,30], type 2 diabetes mellitus [24,31,32], and asthma [33-35]. Because infancy is a critical period of developmental plasticity with long-lasting metabolic and behavioral consequences [36-38], interventions developed for delivery during this period may alter long-term risk for obesity and associated co-morbidities. With 22.8% of 2–5 year old US children already meeting criteria for overweight [39], and overweight by age 5 years strongly associated with later life overweight [40], early interventions to address this epidemic are needed. However, while modifiable factors promoting overweight and rapid growth during infancy have been identified [41-43], until recently, studies aimed at the primary prevention of obesity through infancy-based interventions have not been conducted [44,45].

This paper describes the *I*ntervention *N*urses *S*tart *I*nfants *G*rowing on *H*ealthy *T*rajectories (*INSIGHT*) study, a prospective, two-arm, randomized, controlled trial evaluating the efficacy of a responsive parenting intervention designed to prevent rapid infant weight gain and childhood obesity among first-born infants. The parenting intervention is being compared with a home safety control, in a birth cohort of infants and their parents. The study will follow families until first-borns are at least 3 years old with body mass index (BMI) as the study’s primary outcome. This outcome provides significant insight into long-term obesity risk [40].

INSIGHT’s parenting intervention is grounded in the developmental literature on parenting sensitivities [46,47] and centers on responsive feeding, such that parents are taught how to identify and respond sensitively and appropriately to infant hunger and satiety cues. Such early intervention is hypothesized to positively influence the developing controls of food intake by avoiding controlling, restrictive, or coercive feeding by parents that can attenuate children’s responsiveness to hunger and satiety cues promoting eating in the absence of hunger, preferences for energy dense foods, and increased obesity risk [48,49]. INSIGHT’s central hypothesis is that responsive feeding promotes self-regulation and shared parent–child responsibility for feeding, reducing risk for overeating and overweight [50].

In this study, nurses deliver interventions to first-time parents and their infants in both study groups at four home visits in the first year after birth followed by annual clinical research center visits at age 1, 2, and 3 years. The parenting intervention teaches first-time parents to interact with their infants in a way that is prompt, emotionally supportive, contingent, and developmentally appropriate. Behavioral states (alert and awake, fussy, drowsy, sleeping) serve as a foundation for messages as several portions of the intervention focus on transitioning infants out of the fussy state and into either the alert and awake or the sleeping state with appropriate methods. Behaviors were chosen for our intervention from each of these states that are modifiable, linked to obesity risk, and contain lifestyle benefits for parents that will serve as motivation for behavior change. These additional benefits make the obesity prevention goal “stealth” as described by Robinson [51].

INSIGHT differs from current practice which typically focuses on nutrition sufficiency during the first months after birth. Instead, INSIGHT recognizes that feeding is commonly used as a routine first response to infant and toddler distress and instead promotes parental responsiveness to their child’s needs [52-54]. When feeding is responsive to children’s needs, shared responsibility in feeding begins to develop. As in other areas of development, this provides opportunities for self-regulation as children assume an increasing role in determining when and how much to consume. For future clinical application, this approach has the advantage of allowing healthcare providers to promote positive parenting behaviors associated with responsive feeding as opposed to the more negative theme of prevention of obesity and its co-morbidities.

The framework of responsive parenting underlies the specific lessons in each of the four behavioral states, including instructing parents: a) to recognize infant hunger and satiety cues as well as use feeding more selectively in response only to hunger, b) to use alternatives to feeding to soothe a fussy, but non-hungry infant and toddler, c) to provide children appropriate portions of healthy foods and allow children to determine the amount consumed, d) to improve acceptance of developmentally appropriate foods such as vegetables by using repeated exposure and positive role modeling, e) to develop good sleep hygiene and f) to actively engage infants in play time in order to reduce sedentary behaviors. In addition to these messages, intervention parents are given education on growth charts, the meaning of growth chart percentiles, and healthy growth patterns during early life.

### Obesogenic parenting and the need for parental responsiveness today

Human biology evolved to promote survival in the context of food scarcity, biasing us to eat opportunistically and lay down fat stores essential for survival in times of scarcity. Within an ecological framework, parents protect their children from these perceived environmental threats to protect their health and development [55], and traditional feeding practices evolved during times when food scarcity and disease were threats to child health. Because loss of appetite is often a symptom of child illness and because adequate food and fluid intake is critical to child survival, traditional feeding practices are not contingent on child hunger, and feeding is the “default” response to infant distress [56-58]. Accordingly, traditional feeding practices include strategies intended to soothe infant distress and promote intake, growth, and health such as use of feeding as the first response to crying and other distress, offering preferred foods, promoting intake in the absence of hunger, offering large portions, coercing children to eat beyond satiety, and providing palatable, preferred foods [52-54].

Today in the US and many other countries worldwide, the obesogenic environment promotes an excessively positive energy balance, overweight, and obesity, and too much food has become the major environmental threat to child health, yet traditional feeding practices remain in place. Further, the use of feeding to soothe, especially with infants who are high in negativity and cry frequently, can exacerbate the effects of obesogenic environments, promoting overfeeding and overweight and fostering maladaptive eating behaviors affecting intake and obesity risk throughout the lifespan [54]. These eating behaviors include problems in controlling food intake: increased responsiveness to food cues [59-61], reduced responsiveness to hunger and satiety, disinhibited eating and eating in the absence of hunger, and excessive weight gain and obesity [49,62].

### Current clinical care does not promote responsive feeding of infants, shared parent-infant feeding responsibility, and healthy diet content

Currently, clinicians pay limited attention to obesity prevention during infancy [63]. While infant growth is monitored and most clinicians promote breastfeeding, suggest avoidance of infant cereal in bottles, and advise against early introduction of complementary foods and fruit juice, guidance aimed at early life obesity prevention often stops there. While initially appropriate, the focus for newborns and infants is typically on promoting adequate weight gain rather than preventing excessive weight gain. For example, immediately after birth mothers are typically instructed to wake their infants to feed at least every 3–4 hours for several weeks [64]. While this is necessary initial advice to help newborns regain weight lost shortly after birth, clinicians are far less consistent in instructing parents when to stop this practice and how to identify and rely on infant hunger and satiety cues to guide feeding. Typical anticipatory guidance in pediatric healthcare does not discourage the use of feeding to soothe for non-hunger related infant distress nor does it advise against feeding as a reward for positive behaviors later in infancy and childhood unless obvious overweight or obesity develops.

Current clinical care does focus on timing of introduction of foods – solids at 4 to 6 months, finger foods at 6 months, no cow’s milk until 1 year, etc. While guidance about what and when to feed infants is provided, little evidence-based direction is given on how to feed infants to promote subsequent healthy eating habits, including the aforementioned sensitive feeding styles, as well as feeding practices that promote acceptance of healthy foods and flavors in our obesogenic environment. However, interventions during infancy can produce healthier eating behaviors. For example, it is accepted that infants have innate preferences for sweet and salty tastes and are “neophobic”, rejecting new foods that are not sweet or salty [65]. Given this set of predispositions, typical infants will readily accept sweet and salty foods such as sweetened drinks and French fries. In contrast, healthy foods such as pureed vegetables, meats, infant cereals, and dairy products, which are not high in sugar or salt, are likely to be initially rejected. Birch and colleagues have conducted research demonstrating that infants typically need several opportunities to sample new foods before intake increases [66,67]. The liking for complex flavors that are not dominated by sweet or salty tastes must be learned [65]. Interventions emphasizing repeated opportunities to try healthy, developmentally appropriate foods can have lasting positive effects on acceptance of healthy foods.

Current advice to parents to make healthy food choices for older infants and toddlers is failing to produce the desired outcomes and highlights the significant need for changes to clinical practice. Data from the Feeding Infants and Toddlers Study (FITS) revealed that unhealthy habits start early; energy intakes among infants and toddlers exceeded requirements by 20-30% [68]. In addition to consuming too much energy, children 4 to 24 months old ate significant amounts of developmentally inappropriate foods, high in energy density and sometimes deficient in key nutrients, and consumed too few of the foods that should form the basis of a healthy weaning diet [69,70]. For example, in children aged 7 and 24 months, 18% and 33% consumed no servings of vegetables, respectively, during a given 24-hour period. Twenty-three percent of 7-month old and 33% of 24-month old children did not consume any fruits. By 15 to 18 months, French fries were the most common vegetable consumed. Clearly, parents of infants and toddlers need better direction on what to feed, how much to feed, and how to promote acceptance of a variety of healthy foods during the transition to the modified adult diet. Evidence that these outcomes can be promoted in a real-world setting is critical to transforming how parents view “picky eaters” while promoting acceptance of healthy diets.

### Building on evidence from recent trials

Until recently, studies aimed at primary prevention of obesity through infancy-based interventions had not been conducted [44,45]. However, over the past several years several trials have been completed with additional studies underway (Table 1). The first to demonstrate an effect on infant weight status was our pilot study, The SLeeping and Intake Methods Taught to Infants and Mothers Early in life study (SLIMTIME) [71]. In this trial, which informed the design of INSIGHT, we selected two promising interventions for obesity prevention based on Birch’s research, recruited first-time mothers who intended to breastfeed, and followed their infants delivered at a single center from birth to 1 year. Research nurses delivered interventions in the home using a 2 × 2 randomized, experimental design. The first intervention, “Soothe/Sleep,” began when infants were 2–3 weeks old. This intervention focused on promoting responsive feeding especially at night via techniques that trained parents to use feeding and other soothing approaches appropriately and selectively to calm a fussy infant [72]. The goal was to help mothers learn to discriminate their infant’s hunger from other distress cues, to use appropriate soothing responses for infant distress, and to prolong infant sleep duration. Our hypothesis was that by helping parents respond appropriately and contingently, this would reduce the use of feeding as the traditional “default” response to infant fussing and crying, and that reducing the use of “feeding to soothe” would reduce the risk of overfeeding and overweight. The second intervention, “Introduction of Solids,” focused on the transition to solids, providing information on which foods to offer or limit, information about portion sizes, and strategies for promoting liking and acceptance of healthy complementary foods. Specifically, it was based on Birch’s research showing the effectiveness of repeated exposure at reducing food neophobia and promoting infants’ acceptance of new vegetables [66,67] and toddlers’ acceptance of new table foods. 110 participants completed the year-long study.

**Table 1:** Completed and other ongoing randomized, controlled trials aiming to prevent obesity through during infancy

**Name/trial registry number**	**Framework**	**Sample size**	**Intervention(s)**	**Outcome(s)**
***Completed trials***				
Educational intervention to modify bottle-feeding behaviors [73]	Experiential Learning Cycle for Adult Learning	40	- Group intervention for Women, Infants, and Children (WIC) participating mothers of 1–2 month old formula-fed infants	- No difference in daily formula intake at 4–5 months
			- Increase awareness of satiety cues	- Intervention group had greater weight gain than control between time of intervention and follow-up at infant age 4–5 months
			- Limit bottle size to 6 ounces or less in first 4 months	
First steps for mommy and Me [74]	Motivational Interviewing	84	- Primary care provider “negotiations” at well child care to endorse behavior change	- Later introduction of solids
			- Health educator calls between visits to discuss maternal healthy lifestyle plus infant obesity preventive guidance	- Modestly less TV viewing
			- Printed Materials	- Larger increases in nocturnal sleep duration from baseline to follow-up and improvements in sleep hygeine
			- Monthly group parent training sessions	- No significant difference in weight-for-length z-score
SLeeping and Intake Methods Taught to Infants and Mothers Early in Life (SLIMTIME) Study [71]	Responsive Parenting	160	- 2×2 design using home nurse visits among mothers intending to breastfeed	- “Soothe/Sleep” breastfeeding infants slept more, had fewer noctural and total daytime feeds
			- “Soothe/Sleep” - discriminate hunger vs. other distress, educate on soothing strategies, day/night differences	- “Introduction of Solids” infants – later intro & were more likely to accept novel healthy foods at age 1 year
			- “Introduction of Solids” - delay introduction, hunger/satiety cues education (2–3 weeks), repeated exposure to vegetables (~4-6 months)	- Infants receiving both interventions had a significantly lower weight-for-length z-score at age 1 year
Healthy Beginnings Trial [75,76]	Health Beliefs	667	- Intensive home nurse visitation over first 2 years plus phone support vs. usual care among socially high-risk families	- At age 2 years, BMI significantly lower for intervention group vs. control
			- Key messages: “Breast is best”, “No solids for me until 6 months”, “I eat a variety of fruit and vegetables every day”, “Only water in my cup”, “I am part of an active family”	- Intervention group ate more vegetables, less meals with TV, and more physical activity
NOURISH Trial [77-79]	Cognitive Behavioral with Anticipatory Guidance	698	- Two modules of group parent education and peer support sessions held co-led by dietician and psychologist timed around a) introduction of solids and b) emergence of autonomy and independence	- Lower BMI-for-age Z-score and less rapid infant weight gain since birth at 13–14 months
			- Parents instructed to overcome neophobia and increase healthy food acceptance through teaching on healthy infant growth and requirements, variability of intake within/between infants, amount/timing of snacks, hunger/satiety cues	- No difference with control group for BMI at age 2 years
			- Parents instructed to help develop infant self-regulation and healthy diet with lessons on managing food refusal/neophobia/fussing, developmental need for autonomy and limit testing, modeling healthy food choices	- Mothers used more responsive feeding practices
				- Mothers less likely to use food as a reward or turn meals into a game
MOMS Project [80,81]	Anticipatory Guidance	292	- Primary care anticipatory guidance-based study comparing 3 interventions delivered at well child care by primary care providers plus handouts: Mother focused (maternal eating habits and modeling eating) vs. Infant focused (serving size, introduction of solids, feeding style) vs. usual care	- No difference in growth parameters between groups at 1 year
				- Mothers in mother and infant focused groups gave less juice and gave more fruit and vegetables than those in the usual care group.
The Infant Feeding Activity and Nutrition Trial (INFANT) [82-84]	Parent support theory; Social cognitive theory	542	- Community-based existing maternal-child health nurse-led groups with dietician led intervention (6 – two hour sessions delivered quarterly) vs. control (usual care)	- At age 20 months there was no difference in BMI between groups, but intervention group showed a modest reduction in sweet snack intake and a modest reduction in TV viewing
			- Developmentally appropriate guidance on parent feeding style, timing of introduction of solids, nutrition, parent modeling, managing food rejection	- No group differences in fruit/veggie/water/sweetened beverage intake, physical activity
***Trials with Published Methods***				**Primary Outcome**
Mi Voglio Bene [85]	Anticipatory Guidance	3610	- Primary care based delivery of 10 preventive actions (promotion of breastfeeding, delayed introduction of solids, control of protein intake in first 2 years, avoidance of sweetened beverages, avoidance of bottle use after 2 years, promoting physical activity, identification of early adiposity rebound, limit TV viewing, encouraging play, controlling portion size	- BMI at age 6 years
Prevention of Overweight in Infancy (POI.nz) [86]	Anticipatory Guidance	800	- 4 arm trial comparing usual care with usual care plus either a Food, Activity, and Breastfeeding intervention or a Sleep intervention or both interventions delivered in well care supplemented by research nurses, lactation consultants and/or sleep specialists	- BMI at age 2 years
Healthy Babies [87]	Theory of Planned Behavior	372	- Paraprofessional home visits providing guidance on normative growth and development and skill-building on maternal feeding and feeding responsiveness,	- Weight-for-length at age 1 year
Preventing Childhood Obesity through Early Feeding and Parenting Guidance [88]	Personalized Anticipatory Guidance	140	- Community health worker home visits with focus on preventing obesogenic feeding behaviors, parental recognition of cues, play without screen time, and good sleep hygiene	- Weight-for-length at ages 1 and 2 years
Greenlight Study [89]	Social Cognitive Theory	865	- Low literacy materials delivered during well child visits by pediatric residents focusing on satiety cues, sweetened beverages, introduction of solids, portion sizes, non-sedentary activity, and breastfeeding	- BMI at 2 years

In comparing “Soothe/Sleep” intervention infants vs. control, results showed that breastfed intervention infants slept significantly longer at night 16 weeks after birth (*p* = .04). Compared with controls, the intervention also reduced total number of daily feedings (*p* = .008) and nocturnal feedings (*p* = .003) for breastfed infants. Consistent with our framework, the results suggest that our intervention helped parents learn to be responsive while calming infants without feeding in the absence of hunger, thus promoting parenting competence. It also appeared to promote development of infant self-regulation by allowing infants opportunities to self-soothe [90,91]. Third, given the consistently demonstrated relationship between short sleep duration and overweight, obesity and higher body fat for children of all ages including infants [9,20,43,92-98], an intervention that effectively lengthens sleep duration during infancy is potentially preventive, and may have both short- and long-term effects, given that infant sleep difficulty predicts later sleep problems [99,100].

The “Introduction of Solids” intervention focused on the timing of and effective approaches for introducing solid foods. The intervention delayed introduction of solids, increased acceptance of vegetables upon introduction, and increased acceptance of healthy, unfamiliar foods (hummus, cottage cheese, or yogurt) at the age 1 year laboratory visit. Based on intervention-blinded coding of videos, only 10% of intervention infants rejected an unfamiliar food at age 1 year compared with 25% of those in the control group [71].

In addition to improving secondary outcomes related to sleeping and feeding, participants receiving both study interventions had significantly lower weight-for-length percentiles at 1 year (43rd percentile) than those in other study groups. The findings suggest both interventions, the first affecting early sleep, soothing, and feeding frequency and the second affecting the infants’ reactions to the introduction of solid foods, were required to have a significant effect on weight status, and therefore a multi-component intervention was selected for the larger INSIGHT trial.

## Methods/Design

### Overall study design, recruitment, and randomization

**Figure 1:**
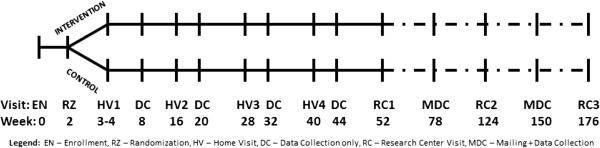
INSIGHT study visit schedule.

INSIGHT is a two-arm randomized, controlled trial that involves nurses delivering interventions to first-time parents and their infants at four home visits in the first year after birth followed by clinical research center visits at ages 1, 2, and 3 years (Figure 1). Following completion of informed consent, research staff collected data from the newborn medical record, and participating mothers completed baseline demographic questionnaires. Because we are interested in the effect of the intervention program on formula fed and breastfed infants, mothers were contacted via telephone 10 to 14 days following childbirth, and randomized to a study group with stratification performed based on mothers’ intended feeding mode (breastfeeding or formula) and sex-specific birth weight for gestational age (<50th percentile or ≥50th percentile) [101]. This study was approved by the Penn State College of Medicine’s Human Subjects Protection Office and registered at http://www.clinicaltrials.gov (NCT01167270) prior to enrollment of the first participant.

### Participants

**Figure 2:**
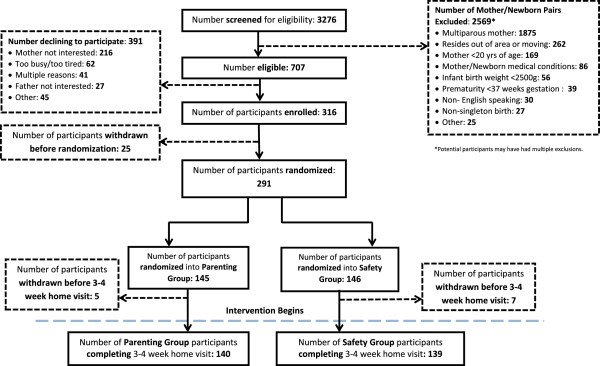
Study CONSORT diagram.

All newborns delivered at the Penn State Milton S. Hershey Medical Center in Hershey, Pennsylvania were screened for participation (Figure 2). Eligible mother-infant dyads for this trial included full-term (≥37 weeks gestation), singleton newborns delivered to English-speaking, primiparous mothers ≥20 years of age residing within 50 miles of the center. Mother-newborn dyads were excluded if there was a plan for the newborn to be adopted or move from Central Pennsylvania within 3 years, if a prenatal ultrasound demonstrated evidence of intrauterine growth retardation, if the newborn’s birth weight was <2500 grams, or if either the mother or newborn had significant health issues that would affect study participation.

### Sample size

The primary outcome for INSIGHT is BMI Z-score at 3 years. A 0.67 difference in BMI *Z*-scores represents the distance between major centile lines displayed on infant growth charts. This difference is clinically meaningful, and upward percentile crossing of this magnitude has been the operational definition frequently used for rapid weight gain [102]. For this reason we have powered the study to detect a 0.67 difference in BMI *Z*-scores between the safety control and the parenting intervention groups within each of the feeding modes (intent-to-breast feed or formula feed). To detect this difference with 90% power and a 5% Type 1 error rate, 276 participants are required. This includes an anticipated 30% attrition rate. INSIGHT’s initial sample size slightly exceeded this 276 with a total of 316 mother-infant dyads enrolled, 291 dyads randomized, and 279 participants completing the 3–4 week study visit where the interventions begin. This cohort of 279 will be considered the study cohort for outcomes and analyses.

### Intervention group

**Figure 3:**
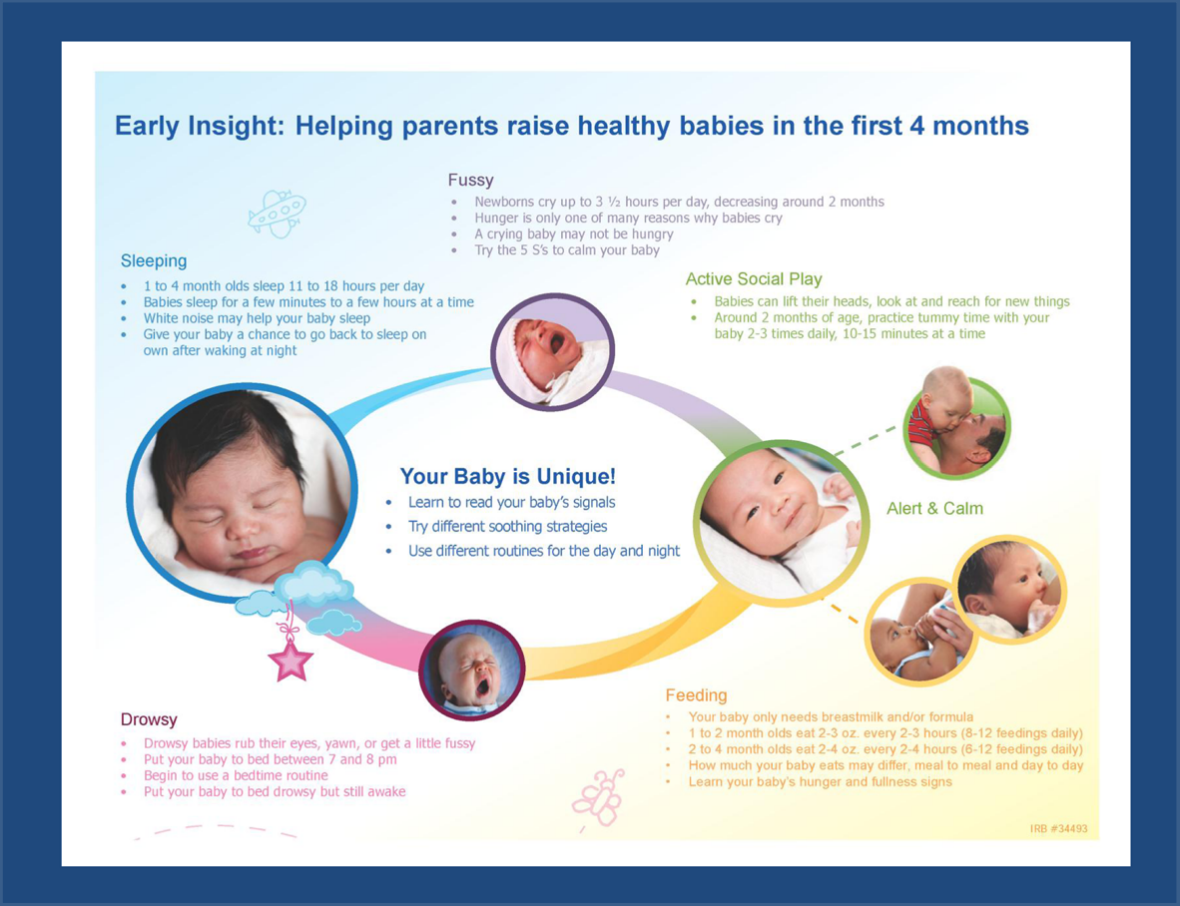
Example of responsive parenting messages delivered for behavioral states.

The parenting intervention uses a responsive parenting framework with obesity prevention messages delivered at each visit that correspond to four infant/toddler behavior states: Drowsy, Sleeping, Fussy, and Alert and Calm (Figure 3). Within the Alert and Calm category are two sub-categories, Active, social play and Feeding.

#### Drowsy and sleep

Beginning at the first study home visit and at each subsequent visit, age appropriate sleep hygiene to promote longer sleep duration is discussed. Norms for sleep duration at study visit are discussed, as are strategies to prolong sleep duration. Avoidance of feeding to sleep is a consistent theme as is recognition of how to respond to night wakings without feeding when appropriate based upon norms for intervals between feedings and hunger and satiety cues. More specific guidance was given at developmentally appropriate ages such as the use of swaddle blankets, white noise, and “dream feeds” (parent waking baby to feed before parent goes to bed) during early infancy, as well as how to transition from multiple to single daily naps, establishing a consistent bedtime routine, and when to transition from a crib to a bed for toddlers. Importantly, parents were given individualized feedback based on their infant’s sleep hygiene by completing a “Sleep Profile” at 16 and 40 weeks based on the work by Mindell and Sadeh [103]. This feedback allowed parents to both understand how their infant’s sleep compares with others at the same age, and also provides tailored strategies to improve sleep duration and reduce nocturnal awakenings.

#### Fussy

Much of the responsive parenting guidance for the fussy child involves not using food to soothe, but rather emphasizing that food is for hunger, not to soothe fussiness or conversely to reward for good behavior. Additionally, the concept of temperament is introduced to parents to enable them to understand why their baby might be different from others, especially with regards to fussiness. To empower parents with alternative strategies to feeding to calm their infant, the parents are given *The Happiest Baby on the Block* video [104] in the first weeks following delivery, and the video’s calming strategies are demonstrated at the first home visit [105]. Expectations for amounts of daily crying at different points during infancy are also detailed. Later in infancy, expectations for stranger anxiety are discussed, as well as using modeling and emotion coaching to promote healthy emotional development, including the self-regulation of emotions. After age 1 year, strategies are reviewed on how to prevent and handle temper tantrums with *The Happiest Toddler on the Block* video [106] used for demonstration.

#### Alert and calm - feeding

Beginning during early infancy, instructions are given to parents on recognition of infant hunger and satiety cues, appropriate portion sizes, using food for hunger only and not for soothing, reward, or punishment, healthy nutrition for children, and modeling of healthy eating by parents. Initially, this focuses on breast milk or formula with delaying introduction of solids foods, but later guidance discusses how to introduce vegetables, fruits and other solid foods to a developmentally ready child with a focus on overcoming neophobia for those foods that are neither sweet nor salty. As the infant gets older, the concept of shared feeding responsibility is highlighted repeatedly and parents are taught to understand their role in providing healthy choices for their child, and that the child’s role is to decide how much to eat based on their hunger and fullness cues. Beverage consumption is a focus throughout the infant and toddler period with concrete recommendations to limit fruit juice consumption to 4 ounces daily with strict avoidance of sugar sweetened beverages as well as how and when to wean a child from a bottle to a cup. The use of portable sippy cups is discussed with a focus on avoidance of frequent drinking of caloric beverages (e.g. milk) at non-meal times. Parents are educated on setting routines and limits around what and when foods are served. At several points, portion sizes for meals as well as snacks are demonstrated via pictures and plastic food replicas, comparing them to an appropriate adult portion pictorially. Picky eating and strategies to overcome this common phenomenon is discussed as the child exerts the typical independence of a toddler.

#### Alert and calm – active, social play

Developmentally appropriate physical activity, parental modeling of behavior, and limit setting (e.g. through consistent discipline, avoidance/limiting screen time) are consistent themes in this sub-domain of the INSIGHT intervention. Daily physical activity is encouraged from the beginning of the study through tummy time and parents playing on the floor with their infant each day. When appropriate, outdoor play is specifically encouraged as is parental modeling of exercise. Examples of games and activities where children directly interact with their parents are explicitly described. Avoidance of television, especially during meals, is encouraged with limited screen time after age 2 years allowed though still never during meals.

#### Growth chart education

During early infancy, parents are educated on typical patterns of growth and weight gain including factors such as nutrition and genetics that contribute to growth. Beginning at the 16 week home visit, color-coded growth charts are shared with parents similar to those used with older children [107]. These growth charts are used as a foundation for discussions about the definition of percentiles and healthy patterns of growth with tailored feedback based on the individual child’s anthropometrics. One specific message communicates that higher percentiles on the growth chart are not desirable in the way that they are for school performance.

### Control group

INSIGHT’s control group receives a developmentally appropriate home safety intervention also delivered by visiting nurses. The home safety visits are designed to be equal in length and intensity to the parenting intervention visits and to avoid messages that could impact energy balance. The home safety intervention also is designed within the framework of the four behavioral states. Within the Drowsy and Sleep domains, prevention of Sudden Infant Death Syndrome is initially the focus with carbon monoxide poisoning prevention and bedroom childproofing topics covered later. Within the Fussy category, strategies to prevent Shaken Baby Syndrome and child abuse are discussed initially followed by avoidance of distracted driving, treatment of fever and other first aid remedies, and avoidance of physical force with discipline. Food safety is a repeated theme in the Alert and Calm – Feeding sub-domain beginning with breast milk and formula handling and storage, progressing to baby food handling, food allergens, choking hazards, and safe storage of food sent to childcare. High chair and stove safety issues are also discussed. For the Active, Social Play component, the first topics covered include fire, bath, and car seat safety. Prevention of falls, poison prevention, furniture safety, and toy safety are all covered within the first year. Gun safety and seasonal topics (insect repellant, sunscreen, trick-or-treating, holiday decorations) are then covered with pedestrian safety, booster seat transition, and hand washing.

### Measures

To assess intervention impacts on both the primary outcome and intermediary behavioral processes, INSIGHT data collection includes measures divided into the following categories: Anthropometrics and Biological Specimens, Infant/Child Behavior, Parenting, Maternal Psychosocial Variables and Behavior, Family Context, and Background/Demographics/Covariates. As detailed in Table 2, many of the measurements are assessed repeatedly.

**Table 2:** INSIGHT study measures

**Construct**	**Time points (child age in weeks)**														
	**0**	**2**	**4**	**8**	**16**	**20**	**28**	**32**	**40**	**44**	**52**	**78**	**104**	**130**	**156**
*Anthropometrics and Biological Specimens*															
Child weight and length/height	**X**		**X**		**X**		**X**		**X**		**X**		**X**		**X**
Mother weight	**X**						**X**				**X**		**X**		**X**
Mother height, Father weight/height	**X**														
Child DNA (blood, cheek swabs)											**X**				
Child stool microbiome													**X**		
*Child Behavior*															
Sleep [108,109]		**X**			**X**				**X**		**X**			**X**	**X**
Dietary intake [110,111]*		**X**			**X**		**X**		**X**		**X**		**X**		**X**
Temperament [112,113]					**X**						**X**		**X**		
Reaction to foods	← **X** →										**X**				**X**
Motor milestones [114]								**X**			**X**				
Appetite [115]										**X**					
Videotaped self-feeding											**X**				
Neophobia [116]											**X**				**X**
Eating behavior [117]														**X**	
*Parenting*															
Feeding to soothe [54]*		**X**		**X**	**X**			**X**		**X**		**X**		**X**	
Infant feeding mode [118]		**X**		**X**		**X**		**X**			**X**	**X**		**X**	
Self-efficacy [119,120]		**X**		**X**				**X**			**X**				**X**
Feeding practices & styles [121,122]					**X**		**X**							**X**	
Structure and Control Feeding											**X**		**X**		
*Maternal Psychosocial Variables and Behavior*															
Postpartum depression [123]			**X**						**X**						
Restrained/disinhibited eating [124]					**X**										
Eating habits [125-127]*					**X**			**X**			**X**				
Sleep [128]*					**X**								**X**		
Dietary intake [110,111]*										**X**				**X**	
Trait anxiety [129]												**X**			**X**
Health Literacy [130]															
*Family Context*															
Home environment (observed)			**X**		**X**		**X**		**X**						
Family functioning [131,132]*				**X**			**X**					**X**			
Playtime and activity [133]*				**X**		**X**					**X**		**X**		**X**
TV viewing and family meals						**X**				**X**		**X**		**X**	
Yard and recreational space [134]*									**X**						
Food insecurity [135]	**X**										**X**		**X**		**X**
*Background, Demographics, and Covariates*															
Demographics and Health [118]*	**X**			**X**		**X**					**X**		**X**		**X**
Development knowledge [136]*		**X**	**X**		**X**		**X**		**X**						**X**

*Indicates modified measure – need confirmation that correct ones modified.

### Second child study

Beginning with Sir Francis Galton’s *English Men of Science* published in 1874, researchers have examined relationships between birth order and outcomes related to health, achievement, behavior, and intelligence [137-139]. Yet despite observations over the past 140 years regarding birth order, few have prospectively evaluated differences in parenting between successive siblings as a source of the disparities between first- and second-born children. What is established is that while mothers spend more time interacting with their first-born during infancy [140,141], they are more likely to use restrictive or coercive parenting strategies as first described by Lasko in 1954 [142]. Hilton later found that first-time mothers are significantly more interfering, extreme in response, and inconsistent with parenting response than mothers with their later born children [143]. These findings suggest that parents are less likely to use responsive parenting practices with their first-borns than with later-borns.

A recent review of studies examining the effect of birth order on parenting by Kaley et al. [144] reports that evidence is scant and that to date, no studies have prospectively examined differences in parenting of first- and second-born siblings within the same family beginning at birth. However, the review identified several potentially modifiable postnatal factors affecting infant obesity risk: sleep duration, feeding style, and parental regulation of distress.

Capitalizing on the infrastructure and extensive data collection occurring as part of INSIGHT, a second-related project, SIBSIGHT, adds two major pieces by enrolling second born siblings of INSIGHT participants and collecting genetic specimens from both siblings and their parents. Specifically, this translational research is a) prospectively evaluating obesity-related parenting similarities and differences as well as weight-related outcomes between first and second-born siblings, b) exploring how genetic differences among siblings that are associated with appetite, temperament, and obesity susceptibility affect parent–child interactions, degree of responsive parenting, and weight status, and c) determining whether INSIGHT study intervention carryover effects occur among families participating in the observation-only second-born child evaluation.

Data from diverse cultures have shown that first-born children have a higher risk for obesity [145-152] despite the fact that pregnancy related risk factors for childhood obesity (high pre-pregnancy body mass index, high gestational weight gain, occurrence of gestational diabetes, high birth weight) are more common during pregnancies with second-born children. This suggests that postnatal factors related to parenting are the cause of the disparity between first and second born children’s obesity risk. Using the conceptual framework of responsive parenting, it can be hypothesized that mothers have improved responsiveness and more appropriate caretaking behaviors due to the experience gained with their first child. This hypothesis will be tested by comparing the >100 second-born siblings that can be expected to be born during the funding period with their older siblings with the additional goal to explore how differences in genetic susceptibility to obesity and observed differences in appetite and temperament moderate associations between responsive parenting and weight status at age one year. Data collection for second born children is similar to that demonstrated for the first born in the first year after birth with added aims assessing a) the relationship between infant oral and gut microbiome with weight outcomes and b) the role of epigenetics on obesity development as determined from placenta, cord blood, and peripheral blood obtained at 1 year.

## Discussion

There are few proven prevention strategies available to combat the obesity epidemic. With increasing evidence suggesting the importance of early life experiences on long-term health trajectories, the results of the INSIGHT trial have the ability to inform future obesity prevention efforts in clinical settings. The responsive parenting framework grounded in the developmental literature dovetails nicely into aspects of pediatric care that are already present, but require modifications in the current obesogenic environment.

## Competing interests

The authors declare that they have no competing interests.

## Authors’ contributions

IMP and LLB led all aspects of the study concept and design. JSW, SAF, JSB, KDM, MEM, LBH, SER, NV, and JAM all made substantial contributions to portions of the study design. All authors have been involved in the critical revision of the manuscript and have given final approval to the submitted version.

## Pre-publication history

The pre-publication history for this paper can be accessed here:

http://www.biomedcentral.com/1471-2431/14/184/prepub
